# Use of the Composite Properties of a Microwave Resonator to Enhance the Sensitivity of a Honey Moisture Sensor

**DOI:** 10.3390/s21072549

**Published:** 2021-04-06

**Authors:** José R. Reyes-Ayona, Eloisa Gallegos-Arellano, Juan M. Sierra-Hernández

**Affiliations:** 1 Telecommunication and Photonics Group, Electronics Department, Engineering Division of the Salamanca-Irapuato Campus, University of Guanajuato, Salamanca, Guanajuato 36885, Mexico; jr.reyes@ugto.mx (J.R.R.-A.); jm.sierrahernandez@ugto.mx (J.M.S.-H.); 2 Mechatronics Department, Technological University of Salamanca, Salamanca, Guanajuato 36766, Mexico

**Keywords:** microwave sensor, reflectivity, permittivity, sensor applications

## Abstract

A moisture sensor based on a composite resonator is used to measure different honey samples, which include imitation honey. The sensor changes its frequency response in accordance with the dielectric permittivity that it detects in the measured samples. Although reflectometry sensors have been used to measure the percentage of moisture in honey for almost a century, counterfeiters have achieved that their apocryphal product is capable of deceiving these kinds of sensors. Metamaterial features of the composite resonators are expected to improve their response when detecting lossy samples such as organic samples. It is also sought that these sensors manage to detect small differences not only in the real parts of the dielectric permitivities of samples but also in their imaginary parts, and, thus, the sensors are able to discern between real honey and slightly altered honey. Effectively, not only was it possible to improve the response of the sensors by using lossy samples but it was also possible to identify counterfeit honey.

## 1. Introduction

The measuring of moisture content in honey is quite important for beekeepers. Not only so that they can sell/import their product with the required quality criterion of 17–18%, but also to prevent fermentation in the future because of a high moisture content. The determination of moisture in honey by its refractive index is not new, and it has been known since almost a century ago [1]. Since then, various techniques have been used to determine the percentage of moisture in various materials among which are coil [2], soil [3,4], nuts and grain [5], oil palm fruits [6], foliage [7], and rice grains [8]. A microwave resonator has also been used to measure the moisture in polymers [9]. Moisture sensors at optical frequencies measure the refractive index of the samples to indirectly determine their moisture content [1]. At low frequencies, the electrical impedance [3,5] or the magnetic susceptibility of the samples are measured [8]. In the microwave regime, the reflection coefficient [4,6,9] or the transmission coefficient [7] are used to determine the relative dielectric constant of the samples and, later, this permittivity value is associated with a moisture content amount.

In high-frequency applications, two categories of resonators are used, known as electromechanical resonators and electromagnetic resonators [10]. In the second kind, cavity resonators have a higher-quality factor value but are burdensome and expensive. Dielectric resonators are smaller and cheaper. However, their quality factor *Q* is lower [11]. Finally, transmission-line resonators are the smallest and easily coupled to the circuitry, but they also have the lowest *Q* and they are the least accurate of the three mentioned kinds [11]. A concern with traditional microwave resonators is that, even when they are very accurate, they only work at certain specific frequencies or modes. An additional concern is that its quality factor will be diminished when an external load is applied, to be precise, when a sample to be measured is placed in/on it. After all, the loaded quality factor *Q_L_* of a dielectric resonant structure is always lower than the unloaded *Q*_0_ and external quality factor *Q_e_* because *Q_L_* = *Q*_0_*Q_e_*/(*Q*_0_ + *Q_e_*) [12].

In this work, we aim to use the metamaterial attributes of reconfigurable microstrip resonators [13,14,15] to compensate the dielectric losses of honey. If losses could be compensated, a wider variety of organic samples could be identified by using microwaves. Microwaves could be used to measure the quality of food inside plastic, glass, or cardboard packaging without opening the packaging. Our main goal is to improve the quality factor of our microwave resonators when an organic product is used.

## 2. Sensor Attributes

Whenever dielectric losses are very small, they are usually neglected. If losses are to be considered, the permittivity ε is replaced by *ε*(1 − *j*tan*δ*). The electric field for an ideal cavity is a solution of $\nabla \times \nabla \times E-k_{0}^{2}\kappa E=0$ and of ∇·κE=0 [16]. Such that, when the boundary conditions are satisfied, $k_{0}^{2}$ can be expressed as $k_{0}^{2}=\underset{V}{∭}\nabla \times E\cdot \nabla \times EdV/\underset{V}{∭}\kappa E\cdot EdV.$ If the intensity and direction of **E** and the volume *V* do not have any variations, changes on $k_{0}^{2}$ values are only possible for alterations of the dielectric constant *κ*. The solution is a system of *N* roots or values for $k_{0}^{2}$, which correspond to the eigenvalues for *N* different modes and can be expressed as a function of the guided length of the resonant cavity *l_g_* and of the relative permittivity *ε_r_*.

### 2.1. Resonant Frequencies of the Sensor

An electromagnetic resonator in its simplest model states that its resonant frequency is *f_r_* = 1/(2π√(*LC*)). The associated inductance value L can be controlled and changed by a configuration *D*, and, consequently, the resonant frequency value will also be changed [14]. Thus, the resonant frequency value for our transmission-line-resonator sensor can be expressed as:

(1) $$f_{r}=\frac{c}{l_{g\left(D,N\right)}\sqrt{\varepsilon_{reff}}},$$

where *c* is the speed of light in vacuum, *ε_reff_* is the effective relative permittivity of the sample (from air when no sample is present), and *l_g_*_(*D*,*N*)_ is the guided length of the resonator for the *D* configuration and *N* mode.

**Figure 1:**
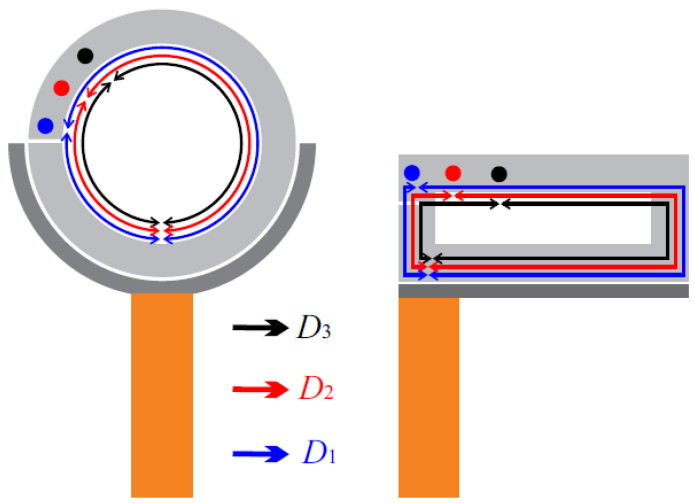
Top view of sensor layouts for circular and rectangular resonators. Orange microstrips are the feeding lines, grey lines are the external coupling lines, and light-grey lines are the resonant structures. Dots display short locations for sensors configuration *D*_1_, *D*_2_, and *D*_3_. Colored arrow-lines are just illustrative to show the difference in distances seen by the traveling waves.

Figure 1 illustrates the top view of microstrip sensors of circular and rectangular resonators. The resonator has a gap on one side to reduce its electrical wavelength and change the impedance seen by the wave on that side. Likewise, the resonator also has a short, denoted by a dot, to force a mode. When a microwave is applied to the sensor, part of the wave will travel on the half side of the resonator and other part of the wave on the right side. Colored arrow-lines are used in Figure 1 to indicate this difference in the distance traveled by the microwaves. By relocating the dot, the traveled distances are changed, and, consequently, the resultant modes can be modified.

**Figure 2:**
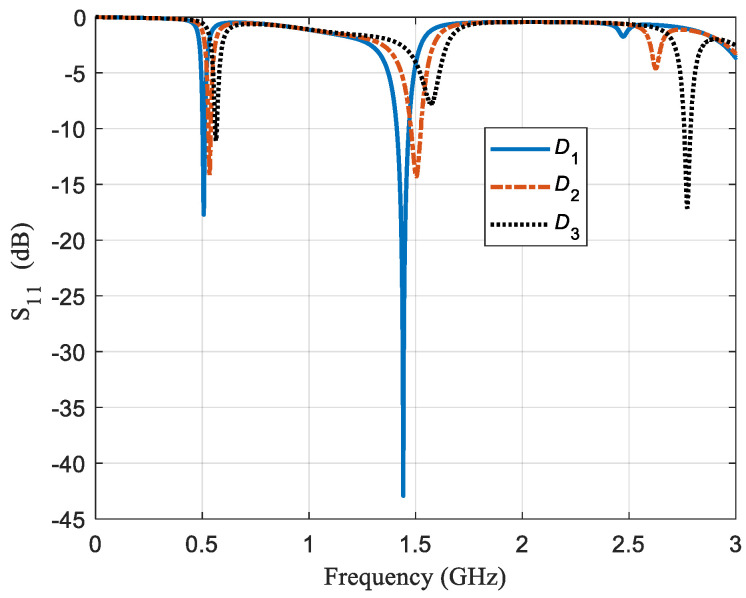
Reflection losses as a function of frequency of the rectangular resonator for three different configurations (*D*_1_, *D*_2_, and *D*_3_). This frequency range shows the three first modes *N* of a resonator at ~0.5 GHz for the first mode, at ~1.5 GHz for the second mode, and at ~2.6 GHz for the third mode. Even though it is the same resonator and modes, reflection loss values are quite different.

Figure 2 presents the frequency response of the sensor for three configurations and the first three modes. For configuration *D*_1_, the second mode resonant is below −40 dB and has a *Q* value of 320, but the third mode resonant is not even below −3 dB and has an inexistent quality factor value. Regarding configuration *D*_2_, there is a small shift in the resonant-frequency values. These values are higher. However, what concerns us is the quality factor values. For the third mode, the *Q* value increases and, for the first and second mode, it decreases, but, for the second mode, it does drastically. Concerning configuration *D*_3_, the qualitative behavior is identical notwithstanding that now the drastic change in the *Q* value is for the third mode. It should be noted that these responses are for the same resonator and sample (air), and that the only change is in the used configuration *D*.

However, if frequency changes are a consequence of the variations of *l_g_*_(*D*,*N*)_ values seen by the applied microwave when *D* is switched, then what is causing the sudden changes in *Q* values? How can these changes be controlled? Finally, and most importantly, how could we use these changes in sample identification?

### 2.2. Dispersion Relation of the Sensor

**Figure 3:**
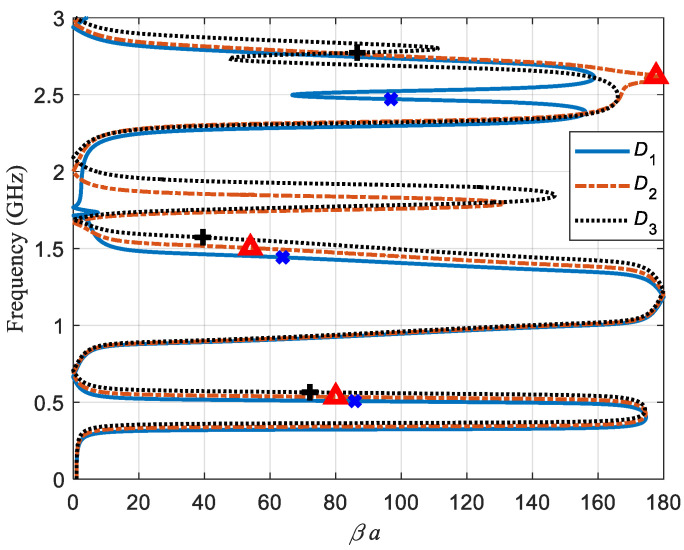
Dispersion relations of the sensor for configurations *D*_1_ (blue solid line), *D*_2_ (red dashed line), and *D*_3_ (black dotted line). Blue x symbols, red ∆ symbols, and black + symbols are placed at the resonant frequency values for the three first modes of configuration *D*_1_, *D*_2_, and *D*_3_, respectively. The dispersion relation reveals forward and backward propagation zones. Between 1.5 GHz and 3 GHz, there are several changes in the direction of propagation and some of them in a very abrupt way, in this zone, which is where the sensor might be more perceptible of small changes in its environment.

Let us first review the nature of the sensor. The resonator is a composite resonator, i.e., the resonator has a right-handed and left-handed behavior. For a frequency range, the resonator’s relative permittivity and permeability are positive and, for the other frequency range, they are negative. Although, all four sign combinations are possible since the operation frequency range is quite wide. Figure 3 depicts the dispersion relation for the three configurations shown in Figure 2.

Dispersion relations were found using the scattering parameters *S*, which were simulated using a full-wave simulator [17], as described in Reference [18]. *βa* is the propagation constant for the sensor. It can be noted that, for some frequency regions, there is a forward propagation and the other propagation is backwards. Blue x symbols indicate the corresponding frequency and propagation constant values for the three modes of the *D*_1_ configuration, as well as red ∆ symbols and black + symbols for *D*_2_ and *D*_3_ configurations, respectively. All shown modes, aside from the third mode of *D*_2_ and *D*_3_ configurations, have a backward propagation. At 1.7 GHz, there is a small flange for the *D*_1_ curve, and the region between 1.6 GHz to 2.1 GHz has no *βa* values bigger than 1.7 of a degree per unit cell. The propagation constant starts as forward propagation, but, suddenly, changes to a left-handed propagation. This also happens for *D*_2_ and *D*_3_, but they reach much higher values of *βa*. These values are 131 and 146 degrees per unit cell.

### 2.3. Input Impedance of the Sensor

**Figure 4:**
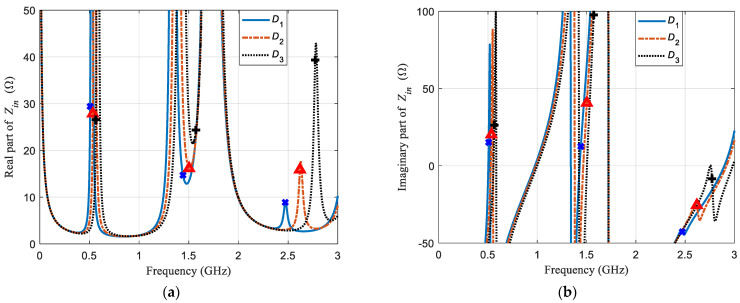
(**a**) Real part, and (**b**) imaginary part as a function of frequency of the input impedance of the sensor for configurations *D*_1_ (blue solid line), *D*_2_ (red dashed-dotted line), and *D*_3_ (black dotted line). Blue x symbols, red ∆ symbols, and black + symbols are placed at the resonant frequency values for the three first modes of configuration *D*_1_, *D*_2_, and *D*_3_, respectively.

Straightaway, let us analyze what the applied microwave would see when it reaches the sensor. More information can be obtained in the examination of the input impedance of the sensor. Figure 4a displays the real part and Figure 4b shows the imaginary part of the input impedance as a function of frequency for configuration *D*_1_, *D*_2_, and *D*_3_. The use of x, ∆, and + symbols is as it was in Figure 3. For the first modes, all real impedance values are below 30 ohms and at a positive sharp slope zone. However, the real impedance values of second modes are almost at a local minimum, and the respective slopes of the location area for each resonant frequency value are: negative for *D*_1_, negative but close to zero for *D*_2_, and positive for *D*_3_. Contrarily, third modes are located at a local maximum zone. All three have a positive slope, but the slope of *D*_1_ is close to zero. It should be recalled that, for third modes *D*_2_ and *D*_3_, are forward propagation modes whereas *D*_1_ is a backward propagation mode.

Lastly, the slope zone of the imaginary part of the input impedance for all six resonant frequencies values of the first two modes is positive and is negative for all three consequent values of the third mode. Furthermore, the smallest amplitude of the imaginary part of the input impedance corresponds to the highest *Q* value for all three modes, and only the values for the third mode are negative. Table 1 shows the input impedance values *Z_in_*, both in their complex form and in their single amplitudes, for the nine resonant frequencies of the three n modes and three *D* configurations.

## 3. Experimental Results

In the previous section, the main features of the sensors were analyzed. At certain regions, a few slight modifications trigger big variations on the response of the sensor. These variations can be made in the configuration of the sensor *D*, which directly affects the guided length of the resonator *l_g_*_(*D*)._ An alternative method to achieve big variations on the resonator response is by modifying the permittivity seen by the resonator since this also modifies its guided length. Although, unlike the variations obtained by the configuration *D*, these modifications can be toward a zone of higher sensitivity or lower sensitivity and this will depend on the specific characteristics of the sample. Therefore, a specific sample that generates a sharp response for one configuration and mode of a sensor could generate a flat response for another configuration or mode of the same sensor. Moreover, a configuration and mode could be excellent for one type of sample but dreadful for another kind of sample. Therefore, several sensors using circular resonators with external diameters of 9, 11, 13, and 15 mm, and a rectangular resonator with long sides of 10, 12, and 14 mm and a width size of 4.4 mm were fabricated. Each resonator microstrip line is 1.8 mm in width. In addition, different configurations were applied to the sensors.

**Figure 5:**
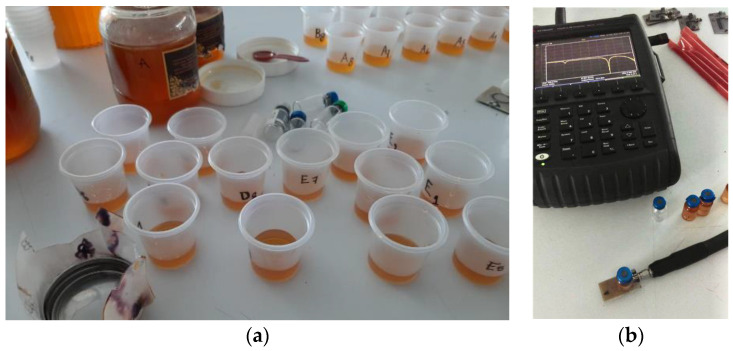
(**a**) A picture of some measured samples. For each of the six available honey varieties, eight samples were separated, weighted, and placed inside a plastic cup. (**b**) A picture of the measurement process where a sample is positioned on top of a sensor. The sensor is connected to a N9914A-21 Vector Network Analyzer (VNA) by means of a low loss precision test cable of the frequency response can be seen on the screen of the VNA.

Six different varieties of honey were measured at room temperature to test the sensors. There are no special conditions inside the laboratory where measurements were performed besides the room temperature that is maintained with a regular air conditioning system. Samples are prepared just before testing to avoid alteration due to the environment since honey is very hygroscopic. Samples are discarded after a few hours ever since their characteristics might not be the same after a while. Three types of honey from different regions of Guanajuato State and one type of imitation honey were provided by The API-EMBE Beekeepers Society from the state of Guanajuato. Another honey sample was supplied by a beekeeper of Texcoco Valley, which is about 400 km from Guanajuato, and a commercial honey brand labeled as 100% pure honey was bought in a convenience store. Eight samples of 7 mL were made for each one of the six honey varieties and placed inside a plastic cup. Sensors were connected by means of Sub-Miniature version A coaxial connectors (SMA connectors) and a precision test cable to a N9914A-21 FieldFox handheld Vector Network Analyzer (Keysigth, Santa Rosa, CA, USA) previously calibrated using the Open-Short-Load calibration technique. Subsequently, each sample was positioned on top of the sensors. Figure 5 exposes a picture of some of the measured samples, as well as a picture of the measurement procedure where a sample is placed on top of a sensor.

### 3.1. Sensor in a Conventional Zone

**Figure 6:**
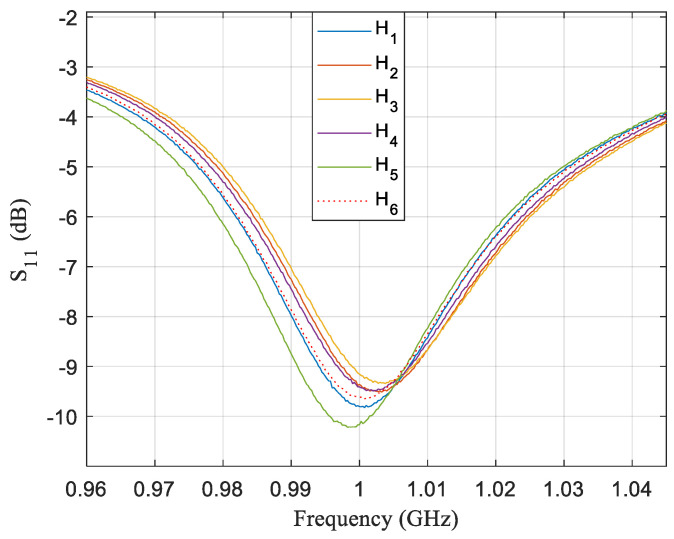
Frequency response of a sensor for a single test where the sensor is configurated in a conventional zone for six honey samples. Frequency responses obtained for the different samples are almost identical, while differences are practically negligible. There is a variation in insertion losses of just 1 dB and in frequency resonance of only 6 MHz at most. In addition, the amplitudes of insertion losses are ~ −9.5 dB. Even though they are acceptable, they also are ordinary.

Figure 6 shows the measured frequency response of a 15 mm circular sensor for the six samples H1–H6 corresponding to six varieties of honey. H6 is the only one with a dashed line since it is the imitation honey sample. Frequency responses do not show significant differences between them yet. The only curve that stands out a little since it is the one and only that is below −10 dB and that is slightly apart from the other curves is the response corresponding to the commercial-brand sample H5. Commonly, commercial brand honeys are a mixture that include honeys from different regions and flowers. To have a uniform mixture, honeys are usually heated, and this heating process could reduce the quality of the mixture [19].

### 3.2. Sensor in Enhancing the Performance Zone

**Figure 7:**
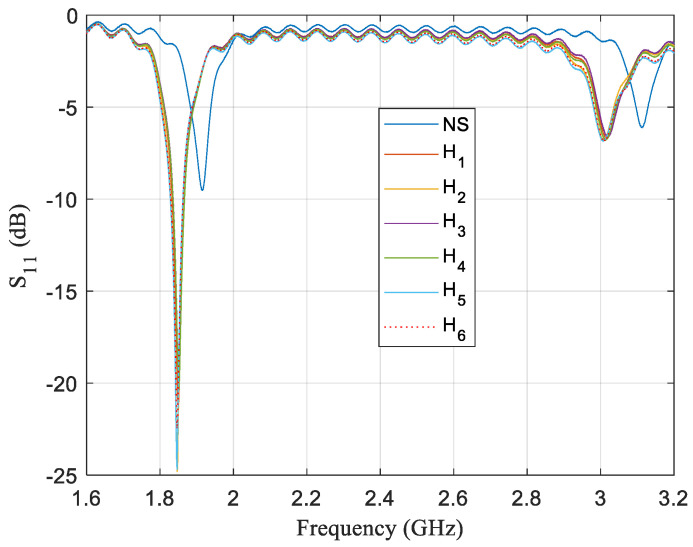
Frequency response of a sensor for a single test where the sensor is configurated in enhancing the performance zone for the same six honey samples used for Figure 6. Two modes are displayed to show off the discrepancies. For the mode ~3.1 GHz, there is almost no change in the insertion loss values when honey samples are present or not. Nevertheless, for the mode of around 1.9 GHz, there is a significant change from −9 to −23 dB. The change in frequency when there is no sample and when a sample is placed on the sensor is consistent with the difference in relative permittivity of air and honey.

The same samples were measured by a 13 mm circular sensor in configuration *D*_1_. This was done to find a better response region for this sort of sample. Figure 7 depicts the sensor frequency response for the second and third mode for the six honey varieties and for the sensor in air that we refer to as No sample (NS). It can be observed that, for the third mode, which is around 3.1 GHz, the curves are typical frequency responses of resonators whose relative permittivity is increased, i.e., these resonances moved to a lower frequency because the sensor detects a bigger relative permittivity value, even though it should be noted that their amplitude increase a little when, for conventional resonators, this amplitude decreases.

Nonetheless, second mode frequency responses of the sensor are quite different when honey samples are present. As can be seen in Figure 7, resonant frequency values decrease when samples are present due to a bigger real part of the relative permittivity. As previously mentioned, this behavior is common in any resonant structure. A higher relative permittivity implies a lower resonant frequency. However, the amplitudes of the resonances are greatly increased, and this is not a common behavior of resonant structures. The *Q* value is associated with the imaginary part of the relative permittivity, and it is a normal behavior that the *Q* value decreases when a load is connected to a resonator, which would be to place a sample on top of the sensor for our case. The increase in the *Q* value presupposes that the dielectric losses of the resonator help to compensate for the dielectric losses of the samples. The alternative stated that the sensor sees, as opposite, the signs of the imaginary parts of the samples´ permittivity and of the imaginary part of the resonator´s permittivity.

### 3.3. Sensor in High-Enhancing Performance Zone

Although, organic samples have been used to enhance the *Q* value of our moisture sensor, the so far used sensor´s modes and settings are still inadequate for identifying imitation honey samples. Thereby, since one objective is to be able to identify imitation honey from real honey, it was necessary to look for other configurations and/or modes.

**Figure 8:**
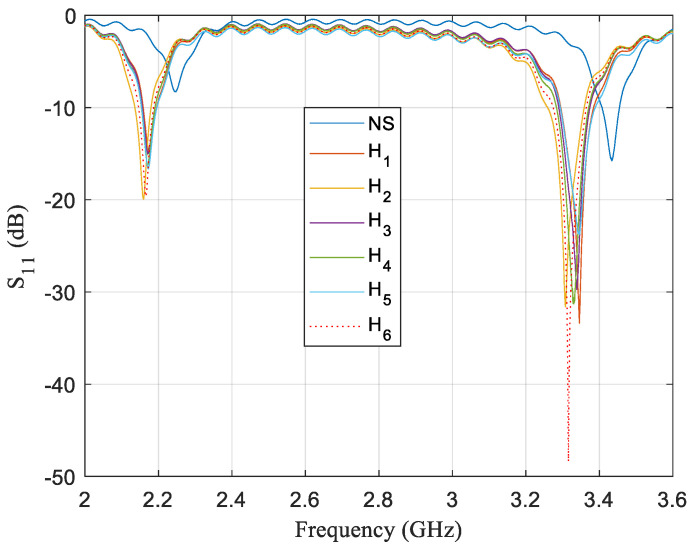
Frequency response of a 13 mm circular sensor for a single test where the sensor is configurated in a highly enhancing performance zone for the same six honey samples used for Figure 6 and Figure 7. Two modes are once again displayed to show off the discrepancies between both modes. For the mode ~2.2 GHz presented on the left side, there is a change in the insertion loss values for No Sample (NS) and honey samples of around 10 dB. For the mode ~3.4 GHz presented on the right side, the difference in insertion loss values is at least of 17 dB for NS and honey samples. In addition, there is a significant discrepancy in the response for the imitation honey sample, which not only has a difference of 30 dB with NS, but also of more than 10 dB with other honey samples.

At long last, it was possible to identify imitation honey from real honey using a 13 mm circular sensor in configuration *D*_2_. Frequency responses where it can be clearly seen that imitation honey has a different response than the other samples are revealed in Figure 8. The reflection coefficient for imitation honey is below −48 dB whereas, for the other samples, their reflection coefficient is close to −32 dB at the most. Five different tests were carried out with the same kind of samples and, in all cases, the reflection coefficient for fake honey is below −44 dB.

## 4. Discussion

Nevertheless, even when it is necessary to carry out tests for each material or food that needs to be identified since their characteristics and, consequently, the responses that the sensor will have are unknown, this sensor could be used to successfully identify samples with small disparities. Therefore, sensors with these attributes will be used to measure and identify several organic samples.

The sensor will be implemented in an identification system that will be provided to the API-EMBE Beekeepers Society so that even persons not specialized in the identification of fake honey can detect apocryphal products.

Besides, this sensor has the advantage that it could be used to identify the quality of liquid or solid food inside plastic, glass, or cardboard containers. Additionally, since it can compensate losses and have a response below −20 dB, it could be used with many organic products.

## 5. Conclusions

The main characteristics of a moisture sensor based on a composite resonator were presented and explained. The properties of the samples were used not only to modify the sensor frequency response but also to improve its quality factor value. We have been able to identify imitation honey from real honey since it was just this honey that would present a response below −44 dB for all its measured samples. The sensor could be used not only to characterize but also to identify samples that seem to be similar by other methods. However, since the properties of the possible samples are unknown, many frequency regions and configurations might be put for testing.

## 6. Patents

The sensor is in a process of patent registration.

## Figures and Tables

**Table 1:** Input impedance values of the sensor in ohms for three *N* modes and three *D* configurations at resonant frequencies.

	*D*_1_		*D*_2_		*D*_3_	
*N*	*Z_in_*	|*Z_in_*|	*Z_in_*	|*Z_in_*|	*Z_in_*	|*Z_in_*|
1	29 + *j*15	33	28 + *j*20	34	27 + *j*26	37
2	15 + *j*13	19	16 + *j*41	43	24 + *j*98	100
3	9 − *j*42	43	16 − *j*25	30	39 − *j*8	40

## Data Availability

The data presented in this study are available on request from the corresponding author.
